# ECG based assessment of circadian variation in AV-nodal conduction during AF—Influence of rate control drugs

**DOI:** 10.3389/fphys.2022.976526

**Published:** 2022-10-04

**Authors:** Mattias Karlsson, Mikael Wallman, Pyotr G. Platonov, Sara R. Ulimoen, Frida Sandberg

**Affiliations:** ^1^ Department of Systems and Data Analysis, Fraunhofer-Chalmers Centre, Gothenburg, Sweden; ^2^ Department of Biomedical Engineering, Lund University, Lund, Sweden; ^3^ Department of Cardiology, Clinical Sciences, Lund University, Lund, Sweden; ^4^ Vestre Viken Hospital Trust, Department of Medical Research, Bærum Hospital, Drammen, Norway

**Keywords:** atrial fibrillation, atrioventricular node, circadian variation, mathematical modeling, genetic algorithm, mixed effect modeling, ECG, rate control drugs

## Abstract

The heart rate during atrial fibrillation (AF) is highly dependent on the conduction properties of the atrioventricular (AV) node. These properties can be affected using *β*-blockers or calcium channel blockers, mainly chosen empirically. Characterization of individual AV-nodal conduction could assist in personalized treatment selection during AF. Individual AV nodal refractory periods and conduction delays were characterized based on 24-hour ambulatory ECGs from 60 patients with permanent AF. This was done by estimating model parameters from a previously created mathematical network model of the AV node using a problem-specific genetic algorithm. Based on the estimated model parameters, the circadian variation and its drug-dependent difference between treatment with two *β*-blockers and two calcium channel blockers were quantified on a population level by means of cosinor analysis using a linear mixed-effect approach. The mixed-effects analysis indicated increased refractoriness relative to baseline for all drugs. An additional decrease in circadian variation for parameters representing conduction delay was observed for the *β*-blockers. This indicates that the two drug types have quantifiable differences in their effects on AV-nodal conduction properties. These differences could be important in treatment outcome, and thus quantifying them could assist in treatment selection.

## 1 Introduction

Atrial fibrillation (AF) is the most common arrhythmia in the world, with a prevalence of 2–4% in the adult population Benjamin et al. (2019), reaching 7% for those aged 65 and above Di Carlo et al. (2019). It is characterized by rapid and irregular contraction of the atria, originating from highly disorganized electrical activity, and associated with an increased risk of mortality, mainly due to stroke or heart failure Hindricks et al. (2021).

The electrical impulses in the atria are conducted via the atrioventricular (AV) node to reach and activate the ventricles. The AV node can block and delay incoming impulses based on its refractory period and conduction delay properties. During AF - when the AV node is bombarded with impulses from the atria - blocking of impulses prevents the heart from racing, but may not be sufficient to maintain a normal heart rate and will still result in significant beat-to-beat variability in the ventricular activation Corino et al. (2015b); Mase et al. (2017).

To remedy this, rate control drugs can be used in order to modify the conduction properties of the AV node. There are two main types of rate control drugs used for AF treatment; *β*-blockers and calcium channel blockers Hindricks et al. (2021). As the name suggests, *β*-blockers block the *β*-receptors in AV node cells, decreasing the effect of the sympathetic nervous system, whereas calcium channel blockers prevent the L-type calcium channels from opening, thereby reducing the conduction in the AV node cells. Both types of drugs have been shown effective in reducing the heart rate during AF Ulimoen et al. (2013). However, the optimal treatment for a given patient is often chosen empirically. Since the two drug types have different physiological effects on the AV node conduction properties, assessing the drug-induced changes in these AV node properties could provide an important step toward personalized treatment. One of the main differences between the two drug types is the effect on the sympathetic nervous system, which can be quantified by the circadian variation in the AV node conduction properties. Furthermore, previous studies have shown a significant difference in the predominant RR interval between day and night, without a difference in dominant atrial cycle length, suggesting circadian variation in the AV node conduction properties Climent et al. (2010).

Conduction properties of the AV node have previously been characterized using mathematical models based on measurements of the electrical activity in the heart Shrier et al. (1987); Billette and Nattel (1994); Sun et al. (1995). Several models of the AV node during AF have been proposed; both based on invasive data from rabbits Inada et al. (2009); Climent et al. (2011) and humans Jørgensen et al. (2002); Mangin et al. (2005); Masè et al. (2012, 2015), and on non-invasive data from humans Corino et al. (2011, 2013); Henriksson et al. (2015). We have previously presented a network model of the AV node capable of assessing the refractory period and the conduction delay of the AV node from 20-min ECG segments Karlsson et al. (2021). However, continuous assessment of AV conduction delay and refractoriness from 24-hour ECG recordings has not previously been performed; such assessment enables analysis of long-term variations in AV conduction properties.

The aim of the present study is to develop a framework for long-term ECG-based assessment of conduction properties in the AV node, and to utilize this framework for analysis of circadian variation and its drug-induced changes in a cohort of 60 patients with persistent AF Ulimoen et al. (2013). To accomplish this, we propose a problem-specific optimization algorithm able to continuously estimate the model parameters from the previously presented network model Karlsson et al. (2021). Furthermore, the uncertainty of the parameter estimates is assessed using a variant of Sobol’s method Sobol (2001), and the drug-induced differences in circadian variation between *β*-blockers and calcium channel blockers on a population level are quantified using a linear mixed-effect model.

## 2 Materials and methods

**FIGURE 1:**
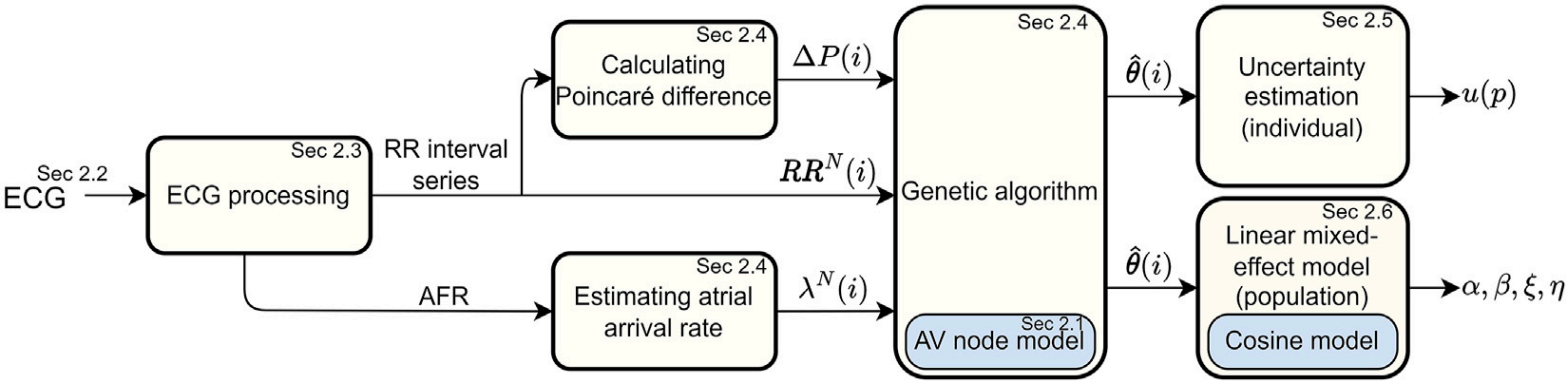
A flowchart of the overall framework for estimating AV node conduction properties on an individual and a population level.

A schematic overview of the methodology is given in Figure 1. The ECG data (Section 2.2) is first processed in order to extract a RR interval series and an atrial fibrillatory rate (AFR) trend, as described in Section 2.3. The RR interval series is then divided into segments of length N, and the AFR trend is used to estimate the atrial arrival rate in the corresponding time interval. The AV node model (Section 2.1) is fitted to the ECG-derived data using a tailored optimization algorithm, as described in Section 2.4, in order to obtain model parameter estimates. Furthermore, the Poincaré plot difference, which quantifies the rate of change of RR series characteristics, is used to tune hyper-parameters in the optimization algorithm during parameter estimation. The uncertainty of the estimated model parameters is investigated using a variant of Sobol’s method, as described in Section 2.5. Finally, cosinor analysis is used to quantify circadian variation in the model parameter trends, and a linear mixed effects modeling approach is used to investigate drug-dependent differences on a population level, as described in Section 2.6.

### 2.1 AV node model

**FIGURE 2:**
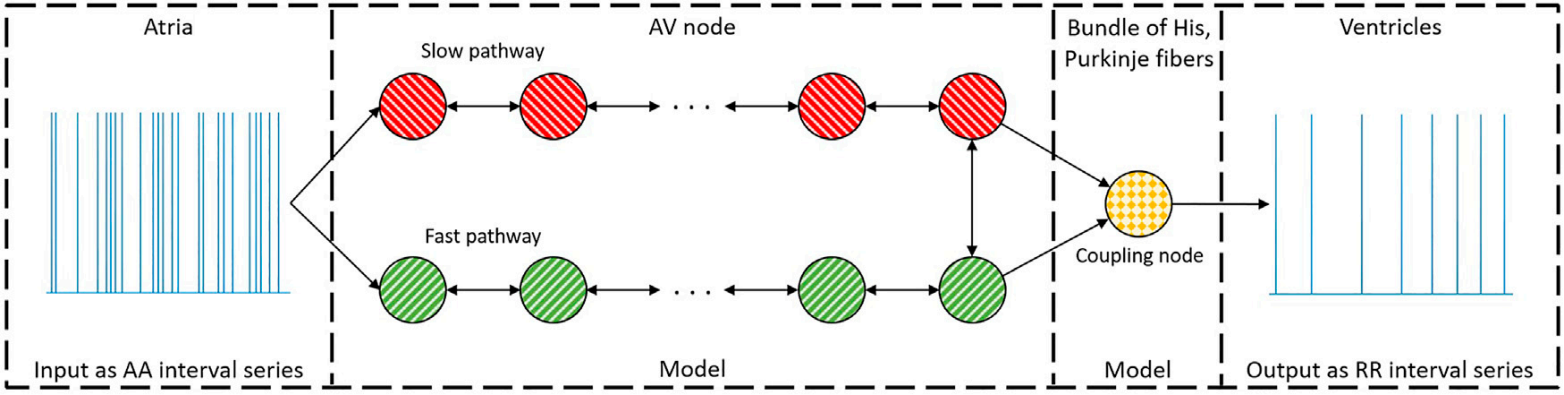
A schematic representation of the network model where the yellow node represents the coupling node, the red nodes the SP, the green nodes the FP, and arrows the direction for impulse conduction. For readability, only a subset of the 21 nodes is shown.

A network model of the human AV node, shown in Figure 2, is used to characterize the conduction delay and refractory period. A brief description of the model is given here, for more details, see Karlsson et al. (2021). The model describes the AV node as an interconnected network of nodes, each capable of transmitting incoming impulses. The model consists of 21 nodes; divided into a fast pathway (FP) with ten nodes, a slow pathway (SP) with ten nodes, and a coupling node. The nodes can react to an incoming impulse either by blocking - if the node is in its refractory state - or by conducting it to all adjacent nodes after adding a conduction delay, after which the node returns to its refractory state. The refractory period (*R*_*j*_(*n*)) and the conduction delay (*D*_*j*_(*n*)) of node *j* following an impulse *n* are given by,

$$R_{j}\left(n\right)=R_{\min}+\Delta R\left(1-e^{\frac{-{\tilde{t}}_{j}\left(n\right)}{\tau_{R}}}\right)$$ (1)

$$D_{j}\left(n\right)=D_{\min}+\Delta De^{\frac{-{\tilde{t}}_{j}\left(n\right)}{\tau_{D}}},$$ (2)

where ${\tilde{t}}_{j}\left(n\right)$ is the diastolic interval preceding impulse *n*,

$${\tilde{t}}_{j}\left(n\right)=t_{j}\left(n\right)-t_{j}\left(n-1\right)-R_{j}\left(n-1\right),$$ (3)

and *t*_*j*_(*n*) is the arrival time of impulse *n* at node *j*. When ${\tilde{t}}_{j}\left(n\right)$ is negative, the impulse will be blocked since the node is in its refractory state. The parameters *R*_min_, Δ*R*, *τ*_*R*_, *D*_min_, Δ*D*, and *τ*_*D*_ are fixed for all nodes in the SP and the FP, respectively. This results in the 12 model parameters $\theta \;=\left[R_{\min}^{FP},\;\Delta R^{FP},\;\tau_{R}^{FP},\;R_{\min}^{SP},\;\Delta R^{SP},\;\tau_{R}^{SP},\;D_{\min}^{FP},\;\Delta D^{FP},\;\tau_{D}^{FP},\;D_{\min}^{SP},\;\Delta D^{SP},\;\tau_{D}^{SP}\right]$ . For convenience, the interpretation of the model parameters are given in Table 1. For the coupling node, the delay is fixed to 60 ms, and the refractory period is fixed to the mean of the ten shortest RR intervals in the data used for model parameter estimation, *RR*_min_.

**TABLE 1:** The interpretation of the model parameters. Superscripts indicating the pathway (SP, FP) are omitted to avoid redundancy.

Parameter	Parameter description
*R*_min_	Minimum refractory period, attained for short diastolic intervals
Δ*R*	Maximum prolongation of the refractory period, attained for long diastolic intervals.
*τ*_*R*_	Time constant for the refractory period, determining the impact of the diastolic interval
*D*_min_	Minimum conduction delay, attained for short diastolic intervals
Δ*D*	Maximum prolongation of the conduction delay, attained for long diastolic intervals.
*τ*_*D*_	Time constant for the conduction delay, determining the impact of the diastolic interval

The input to the model - representing impulses arriving from the atria - is created using a Poisson process with mean arrival rate *λ*. The output of the model represents the time points for ventricular activation, and thus the differences between adjacent elements in the output vector represent the RR intervals.

### 2.2 ECG data

The RATe control in Atrial Fibrillation (RATAF) study Ulimoen et al. (2013) acquired 24-hour ambulatory ECGs during baseline and under the influence of four rate control drugs; the two calcium channel blockers verapamil and diltiazem, and the two *β*-blockers metoprolol and carvedilol. The study population consists of 60 patients with permanent AF, no heart failure, or symptomatic ischemic heart disease. The study was approved by the regional ethics committee and the Norwegian Medicines Agency and conducted in accordance with the Helsinki Declaration. The trend in the AV node refractory period and conduction delay from these five 24-hour ECG recordings per patient is assessed by estimations of the trends in ***θ***.

### 2.3 ECG processing

The RR interval series is extracted from the ECG, where RR intervals following and preceding QRS-complexes with deviating morphology are excluded from the series Lagerholm et al. (2000). Due to excessive noise in the ECGs, some RR intervals are missed, leading to an unrealistically low heart rate. Thus, the data are divided into minute-long non-overlapping segments, and all segments with a heart rate lower than 20 bpm are removed, occasionally resulting in gaps in the signals. The signals with a total duration shorter than 12 h or with less than 20 h between start and end are excluded from further analysis. After excluding data according to these criteria, data from 59 patients remained for inclusion in this study. The number of patients with data considered to be of sufficient duration for analysis and the average duration of these recordings for the different treatments are shown in Table 2.

**TABLE 2:** The number of recordings and recording length (mean ± std) analyzed in this study following exclusion of recordings with insufficient signal quality, as described in Section 2.3.

Drug	Number of recordings	Recordings length (h)
Baseline	51	20.88 ± 2.85
Verapamil	53	21.92 ± 2.39
Diltiazem	56	21.71 ± 2.44
Metoprolol	53	21.87 ± 1.98
Carvedilol	57	21.23 ± 2.65
Total	270	21.52 ± 2.59

The f-waves in the ECG are extracted using spatiotemporal QRST cancellation Stridh and Sornmo (2001). The AFR trends are then estimated by tracking the fundamental frequency of the extracted f-wave signal using a hidden Markov model-based approach Sandberg et al. (2008); resulting in a resolution for the AFR trends of one minute.

### 2.4 Parameter estimation

The atrial arrival rate, *λ*, is estimated by correcting the AFR trend, taking the atrial depolarization time into account Corino et al. (2013). Outliers in the estimated *λ* trends are excluded based on visual inspection guided by cluster analysis. The resulting trends are low-pass filtered using a sliding triangular window filter with a width equal to 70.

The model parameters ***θ*** are assumed to vary over time, making this a dynamic optimization problem. Thus, the data are first divided into overlapping data segments of *N* = 1000 RR intervals; where *N* is chosen to give a good balance between resolution and robustness of the estimates. Each data segment contains one segment-specific mean arrival rate *λ* ^*N*^(*i*) calculated as the mean of the *λ* trend in the segment starting at RR interval *i*, as well as one RR interval series, ***RR*** ^*N*^(*i*). The estimated parameters of a data segment starting at RR interval *i* is denoted by $\hat{\theta }\left(i\right)$ .

A fitness function based on the Poincaré plot - a scatter plot of successive pairs of RR intervals - is used to quantify the difference between observed and simulated RR series. The Poincaré plots are binned into two-dimensional bins with a width of 50 m, centered between 250 and 1800 m, forming a two-dimensional histogram. The error function (*ϵ*), i.e., the inverse fitness function, is then calculated from the number of samples in the bins according to Eq. 4,

$$\epsilon =\frac{1}{K}\overset{K}{\underset{k=1}{\sum }}\frac{{\left(x_{k}^{N}-\frac{N}{N_{\mathrm{sim}}}{\tilde{x}}_{k}^{N_{\mathrm{sim}}}\right)}^{2}}{\sqrt{\frac{N}{N_{\mathrm{sim}}}{\tilde{x}}_{k}^{N_{\mathrm{sim}}}}},$$ (4)

where *K* is the number of bins, *N*_*sim*_ is the number of RR intervals simulated with the model, and $x_{k}^{N}$ and ${\tilde{x}}_{k}^{N_{\mathrm{sim}}}$ are the numbers of RR intervals in the *k*-th bin of the observed data and model output, respectively.

A genetic algorithm (GA) is used to search for the values of ***θ*** yielding the minimum *ϵ*. A GA consists of a population of individuals that evolves based on their fitness value towards a solution using selection, crossover, and mutation Wahde (2008).

By assuming that a large change in the Poincaré plot relates to a large change in parameter values, it is possible before starting the optimization to decide when the optimization algorithm should focus on exploration or exploitation. As a heuristic for this, we introduce the difference in the Poincaré plots (Δ*P*(*i*)), according to Eq. 5,

$$\Delta P\left(i\right)=\frac{1}{K}\overset{K}{\underset{k=1}{\sum }}{\left(x_{k}^{N_{\Delta P}}\left(i\right)-x_{k}^{N_{\Delta P}}\left(i+1000\right)\right)}^{2},$$ (5)

where $x_{k}^{N_{\Delta P}}\left(i\right)$ and $x_{k}^{N_{\Delta P}}\left(i+1000\right)$ are the number of RR intervals in the *k*-th bin of the Poincaré plot for the RR interval series starting at interval *i* and *i* + 1000, respectively. Moreover, the segment length *N*_Δ*P*_ is set to 2000. The Poincaré plot difference, Δ*P*(*i*), is used to tune hyper-parameters in the optimization algorithm.

The GA used for estimating $\hat{\theta }\left(i\right)$ has a population size of 400 individuals - where each individual is a vector of values for ***θ*** - and uses tournament selection, a two-point crossover, and creep mutation Wahde (2008). The number of generations the GA runs before switching to the next data segment varies from 1 when Δ*P(i)* < 800; to 2 when 800 ≤ Δ*P(i)* < 2000; to 3 when Δ*P(i)* ≥ 2000. The step size for the sliding windows is determined by the trade-off between the resolution and the computing cost, and is set to 108 s; resulting in 800 steps for full 24-hour measurements. Thus, there will be 800 estimated $\hat{\theta }\left(i\right)$ for a 24-hour measurement. As noted previously, there are also gaps in the data. Thus, the step size will partly vary to match the start and end of the RR segments, to ensure that all data are used. For reference, estimating the $\hat{\theta }\left(i\right)$ trend from a 24-hour RR and *λ* series using a single core on a standard desktop computer (Intel® Core^*TM*^ i7-6600U Processor, @ 2.60 GHz) requires on average 4 hours.

Since the Poisson process used to create the model input is stochastic, *ϵ* varies between realizations. This variation is dependent on the number of RR intervals generated from the model, where more RR intervals reduce the variation but require more computing power. To have a good balance between computing power and stability, *N*_*sim*_ is set to 1500. However, the ten fittest individuals in each generation are re-evaluated, with *N*_*sim*_ = 5000, before the individual with the best fit for each data segment, $\hat{\theta }\left(i\right)$ , is saved.

The individuals for the first generation are randomly initialized using a latin hypercube sampling in the ranges: $\left\{R_{\min}^{SP},R_{\min}^{FP}\right\}\in \left[150,650\right]\;ms$ ; {Δ*R* ^*SP*^, Δ*R* ^*FP*^} ∈ [0, 700] *ms*; $\left\{\tau_{R}^{SP},\tau_{R}^{FP}\right\}\in \left[40,300\right]\;ms$ ; $\left\{D_{\min}^{SP},D_{\min}^{FP}\right\}\in \left[0,30\right]\;ms$ ; {Δ*D* ^*SP*^, Δ*D* ^*FP*^} ∈ [0, 75] *ms*; $\left\{\tau_{D}^{SP},\tau_{D}^{FP}\right\}\in \left[40,300\right]\;ms$ . These values are also used as boundaries for the model parameters. Hence, the difference between the upper bound and the lower bound for the parameters is the range that the parameters can vary within, here denoted *r*(*p*) and in vector form ***r***, where *p* is the parameter index ordered as in ***θ***.

To reduce the risk of premature convergence and to maintain a good diversity in the population, immigrants - individuals not created from the current population - are used. These immigrants are created using three different methods; 1) by saving and then re-using the ten most fit individuals and their model output per generation; 2) by running eight computationally faster GA, using only 16 individuals and *N*_*sim*_ = 750, simultaneously; and 3), by random sampling. The number of immigrants is dependent on Δ*P*(*i*) and is created in equal proportion using the three different creation methods. These new individuals are then introduced into the population at the start of every new data segment by replacing the individuals with the lowest fitness. More specific details about the GA are found in Supplementary Material, Section 1.

### 2.5 Parameter uncertainty estimation

A variant of Sobol’s method Sobol (2001) is used to derive the uncertainty for each estimated parameter set $\hat{\theta }\left(i\right)$ . The contribution to the output variance (*v*(*p*)) for a parameter *p*, including the variation caused by its interaction with all the other parameters, is estimated by the following procedure. Firstly, two 30 x 12 matrices (***A*** and ***B***), where 30 is the number of sampled parameter vectors, are generated by samples from a quasi Monte Carlo procedure based on the Latin hypercube design. Unlike Sobol’s method - which samples in the whole parameter range - these samples are generated within $\hat{\theta }\left(i\right)\pm 0.075r$ , hence within a hyper-rectangle covering 15% of the total range of each parameter. Secondly, 12 new matrices, ***AB***_*p*_ are created by replacing the *p*-th column in ***A*** with the *p*-th from ***B***. Thirdly, *ϵ* is calculated for each parameter set in the matrices by running the model, before the expected value of the contribution to the output variance is estimated according to Eq. 6 Sobol (2001).

$$\hat{v}\left(p\right)=\frac{1}{2\cdot 30}\overset{30}{\underset{q=1}{\sum }}{\left(\epsilon_{A_{q}}-\epsilon_{AB_{p,q}}\right)}^{2}.$$ (6)

Here $\epsilon_{A_{q}}$ and $\epsilon_{AB_{p,q}}$ quantifies the difference between the observed RR series and the model output as given in Eq. 4, for the parameter sets in ***A*** and ***AB***_*p*_, respectively.

The estimated $\hat{v}\left(p\right)$ are then, together with the mean $\left(\bar{\epsilon }\right)$ and standard deviation (*σ*_*ϵ*_) of the 30 realizations of $\hat{\theta }\left(i\right)$ , used to calculate a parameter uncertainty estimate according to Eq. 7.

$$u\left(p\right)=\frac{0.15r\left(p\right)}{\sqrt{\hat{v}\left(p\right)}-\sigma_{\epsilon }}0.1\bar{\epsilon }.$$ (7)

Here 0.15*r*(*p*) originates from the distance between $\hat{\theta }\left(i\right)$ and the border of the sampled hyper-space, and $\sqrt{\hat{v}\left(p\right)}-\sigma_{\epsilon }$ from the difference between the error variation inside the hyper-space and at $\hat{\theta }\left(i\right)$ . Hence, the fraction relates to the slope-intercept between the parameter distance and the uncertainty. The remaining product relates this slope to 10% of the mean error for $\hat{\theta }\left(i\right)$ . Thus, the interpretation of *u*(*p*) is: ‘Assuming interaction between all model parameters, how large a step can be taken for parameter *p* before the contribution to *ϵ* for $\hat{\theta }\left(i\right)$ is increased by 10%‘. This was then repeated for all $\hat{\theta }\left(i\right)$ for all patients and drugs.

### 2.6 Circadian variation

The drug-dependent circadian variation for the estimated AV node parameters is quantified using linear mixed-effect modeling, i.e., using a statistical model comprising both fixed effects and random effects. The model used consists of a 24-hour periodic cosine with mean ***m***, amplitude ***a***, and phase ***ϕ***, as seen in Eqs. 8, 9, and 10.

$$y_{\mathrm{pat,m}}\left(t\right)=m_{\mathrm{pat,m}}+a_{\mathrm{pat,m}}\;\cos\left(\frac{2\pi }{24}t+\phi \right)$$ (8)

$$m_{\mathrm{pat,m}}=\alpha +\alpha_{m}+\eta_{\mathrm{pat}}+\eta_{\mathrm{pat,m}}$$ (9)

$$a_{\mathrm{pat,m}}=\beta +\beta_{m}+\xi_{\mathrm{pat}}+\xi_{\mathrm{pat,m}}$$ (10)

Here ***y***_*pat,m*_(*t*) represents the estimated parameter trends of patient *pat* during treatment *m* ∈ {Baseline, Verapamil, Diltiazem, Metoprolol, Carvedilol}. Moreover, *t* corresponds to the time of the day, in hours, of the RR interval *i* that the estimated $\hat{\theta }\left(i\right)$ relates to. Furthermore, ***α***, ***α***_*m*_, ***β***, and ***β***_*m*_ represent the fixed-effects; with ***α*** and ***β*** corresponding to the mean value for the mean and amplitude during baseline, and ***α***_*m*_ and ***β***_*m*_ to the average deviation from the baseline values, caused by the drugs. The random effects ***η***_*pat*_, ***η***_*pat,m*_, ***ξ***_*pat*_, and ***ξ***_*pat,m*_ correspond to the individual deviation from the fixed-effects, and are assumed to be sampled from a zero-mean gaussian distribution. During baseline, ***α***_*m*_, ***β***_*m*_ and ***η***_*pat,m*_, ***ξ***_*pat,m*_ are assumed to be zero. For a given individual, *ϕ* is assumed to be equal for all 12 model parameters and is estimated by means of principal component analysis of the $\hat{\theta }\left(i\right)$ trends. The 12 vectors created by projecting the data onto the 12 principal components are fitted to a cosine with mean *m*_*c*_, amplitude *a*_*c*_, and phase *ϕ*_*c*_, where *c* indicates the *c*-th principal component, using the simplex search method Lagarias et al. (1998). The phase, *ϕ*, is set equal to the *ϕ*_*c*_ associated with the highest *a*_*c*_. Moreover, for cases where ***a***_*pat,m*_ is negative, a phase-shift of *π* is added to ensure that all the amplitudes are positive.

With ***ϕ*** estimated, ***α***, ***α***_*m*_, ***β***, ***β***_*m*_, ***η***_*pat*_, ***η***_*pat,m*_, ***ξ***_*pat*_, and ***ξ***_*pat,m*_ are fitted using the linear mixed-effects model function ‘fitlme ()’ in MATLAB (The MathWorks Inc. Version R2019b); using the full covariance matrix with the Cholesky parameterization and the maximum likelihood for estimating parameters of the linear mixed-effects model with trust region based quasi-Newton optimizer as settings.

An assessment of the goodness of fit for the linear mixed-effect model is calculated as the RMSE between the modeled cosine and the estimated parameters. For easier comparison between parameters, the RMSE for each parameter is weighted by their respective range, *r*(*p*).

### 2.7 Statistic analysis

The estimated parameters $\hat{\theta }\left(i\right)$ , as well as AFR and HR, were averaged for each recording, and significant difference between the averages at baseline and under the four drugs were assessed one-by-one using the paired two-sided Wilcoxon signed rank test Woolson (2007) with the Benjamini–Hochberg correction Benjamini and Hochberg (1995). Patients with missing recordings (cf. Table 2) at baseline or the drug in question were excluded from the analysis. A *p*-value below 0.05 after correction was considered significant.

## 3 Results

**FIGURE 3:**
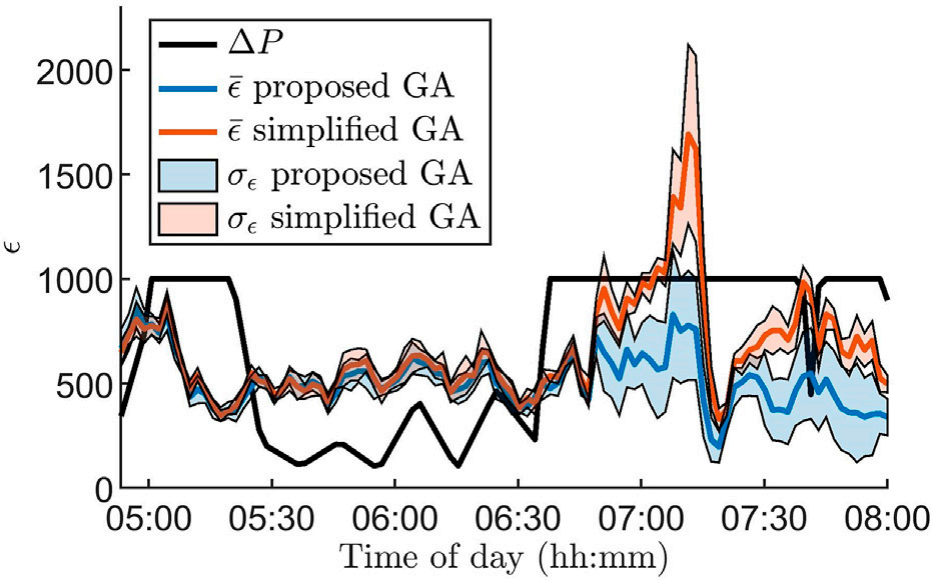
Mean (colored lines) and standard deviation (colored areas) of the error *ϵ* for 100 segments for the proposed genetic algorithm (blue) and a standard genetic algorithm (red) together with the Poincare difference Δ*P*(*i*) (black line), defined in Eq. 5, for data from one patient at baseline during 3 hours. The standard deviation and mean are based on ten runs of the algorithms. Note that Δ*P*(*i*) is scaled with $\frac{1}{5}$ for readability.

Figure 3 illustrates the advantages of using the GA proposed in Section 2.4 for parameter estimation by comparing it to a standard version of the GA. For the standard GA, all hyper-parameters, as well as the number of generations per data segment, are fixed and thus do not take advantage of Δ*P*(*i*). To highlight the differences between the algorithms, we zoom in on a three hour long segment where the RR series characteristics change rapidly. It is clear that *ϵ* increases along with Δ*P*(*i*) for the standard GA, in contrast to the proposed GA.

**FIGURE 4:**
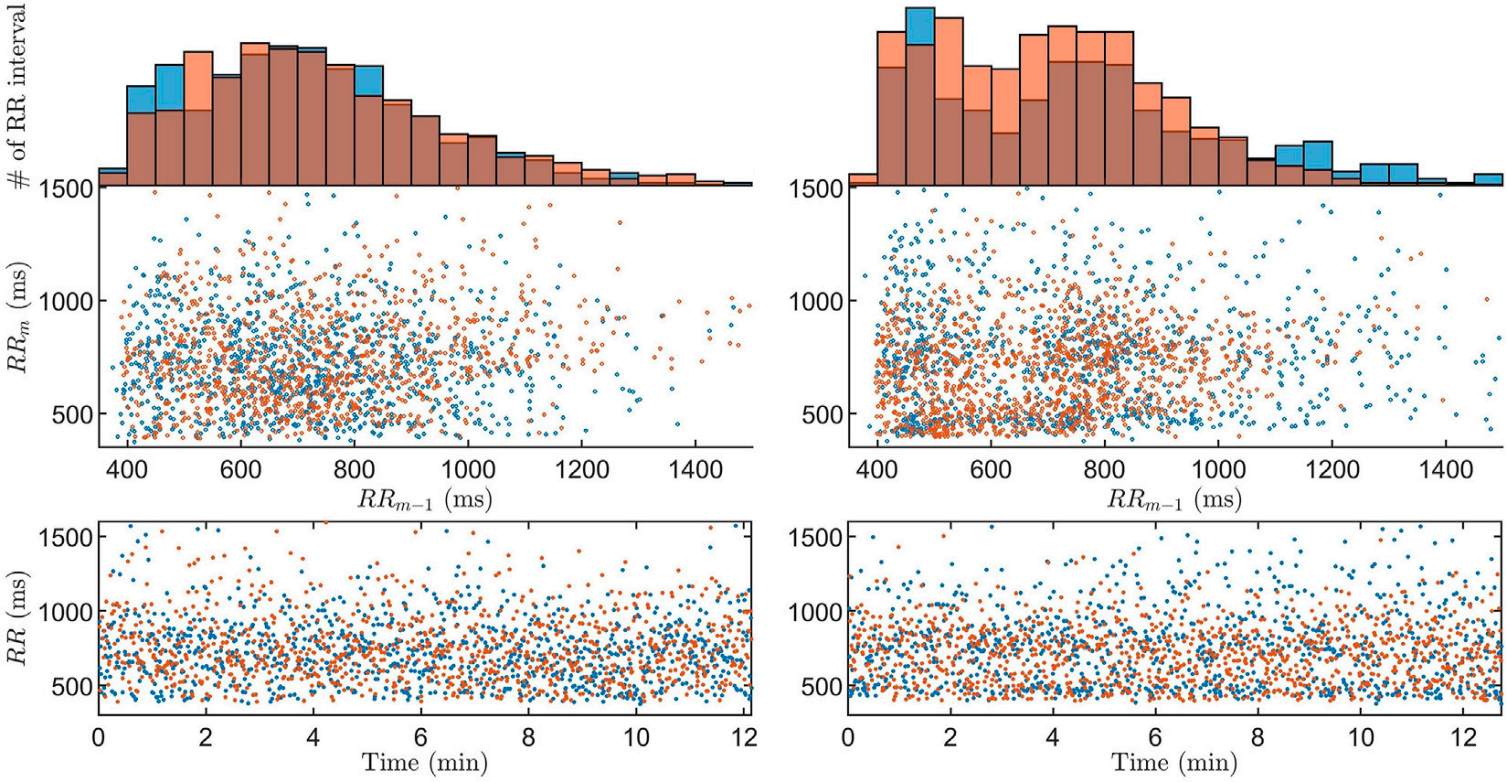
The Poincaré plot with associated histogram and RR interval series of data (blue) and model output (orange) for the $\hat{\theta }\left(i\right)$ corresponding to the median *ϵ* (left) and to the $\hat{\theta }\left(i\right)$ which *ϵ* is higher than 95% of all *ϵ* (right).

**FIGURE 5:**
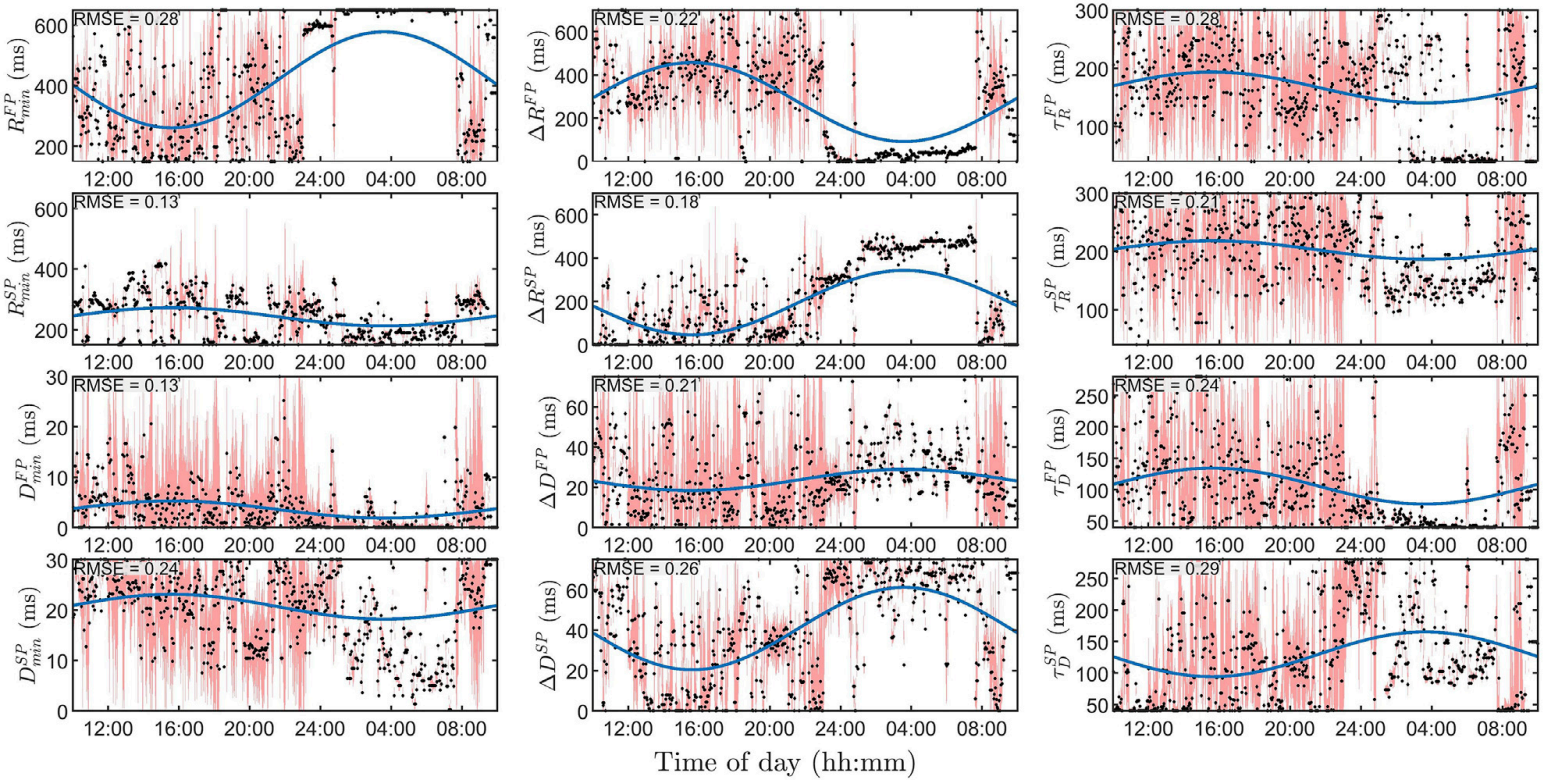
Estimated model parameters $\hat{\theta }\left(i\right)$ (black dots), with corresponding uncertainty estimates *PU* (red areas), along with the fitted cosine (blue lines) used for the circadian variation, for one patient during baseline. In each panel, the RMSE is reported as a measure of goodness of fit between $\hat{\theta }\left(i\right)$ and the fitted cosine. Left column shows the parameters relating to the minimum conduction delay or refractory period, the middle column the parameters relating to the maximum prolongation, and the right column to the time constants. For further explanation of the model parameters, see Table 1.

From the GA we acquire one estimate per data segment, for all 59 patients and all drugs, resulting in a total of 175,640 $\hat{\theta }\left(i\right)$ . To give the reader a sense of the match between the model output and RR interval series obtained from the ECG, we present two examples of Poincaré plots and histograms together with the associated RR interval series. One corresponds to the median *ϵ*, and one where *ϵ* is higher than 95% of all *ϵ*, as shown in Figure 4. It is evident that the histograms and Poincaré plots from the model output and data are similar for both cases, indicating a good match to data in most data segments. However, there is a considerable difference on a beat-to-beat level, as indicated by the RR interval series. Moreover, $\hat{\theta }\left(i\right)$ for one patient at baseline is shown in Figure 5, where clear changes over time can be seen.

Recording averages of estimated model parameters, AFR, and HR at baseline and during treatment with the four different drugs are shown in Table 3. Significant differences, as described in Section 2.7, are indicated in the table by ‘*’. This shows a significant increase in the refractory period in the FP for all drugs, as well as a significant decrease in heart rate and AFR for all drugs.

**TABLE 3:** Recording averages of estimated model parameters, AFR, and HR at baseline and during treatment with the four different drugs (mean ± standard deviation). Differences from baseline are evaluated using the Wilcoxon signed rank test with the Benjamini–Hochberg correction; significant difference from baseline for the drugs, with false discovery rate at 0.05, is indicated with *.

Parameter	Baseline	Verapamil	Diltiazem	Metoprolol	Carvedilol
$R_{\min}^{FP}$ (ms)	435 ± 139	488 ± 134*	518 ± 118*	489 ± 126*	476 ± 123*
Δ*R* ^*FP*^ (ms)	403 ± 195	478 ± 190*	488 ± 202*	495 ± 180*	483 ± 172*
$\tau_{R}^{FP}$ (ms)	175 ± 59	165 ± 63	163 ± 64	162 ± 58	167 ± 57
$R_{\min}^{SP}$ (ms)	241 ± 102	280 ± 125*	287 ± 124*	260 ± 114	269 ± 123
Δ*R* ^*SP*^ (ms)	231 ± 176	274 ± 201	301 ± 215*	312 ± 187*	274 ± 186*
$\tau_{R}^{SP}$ (ms)	180 ± 60	183 ± 62	171 ± 63	176 ± 62	176 ± 63
$D_{\min}^{FP}$ (ms)	5.3 ± 4.5	5.4 ± 4.8	5.4 ± 4.7	5.9 ± 4.5	5.3 ± 4.5
Δ*D* ^*FP*^ (ms)	18.9 ± 16.9	21.7 ± 17.2	22.1 ± 17.3	21.8 ± 16.7	21.4 ± 16.9
$\tau_{D}^{FP}$ (ms)	141 ± 54	144 ± 50	145 ± 53	149 ± 50	142 ± 53
$D_{\min}^{SP}$ (ms)	21.0 ± 5.3	21.6 ± 5.1	22.5 ± 5.2*	21.7 ± 4.8	21 ± 5.2
Δ*D* ^*SP*^ (ms)	26.3 ± 21.4	23.8 ± 20.9	19.6 ± 20.7*	22.6 ± 21.2	21.5 ± 20.8
$\tau_{D}^{SP}$ (ms)	185 ± 68	184 ± 57	183 ± 65	186 ± 58	180 ± 65
HR (bpm)	95 ± 13	80 ± 12*	74 ± 10*	81 ± 10*	84 ± 11*
AFR (Hz)	4.96 ± 0.34	4.56 ± 0.45*	4.71 ± 0.44*	4.86 ± 0.40*	4.81 ± 0.51*

### 3.1 Uncertainty estimation

**FIGURE 6:**
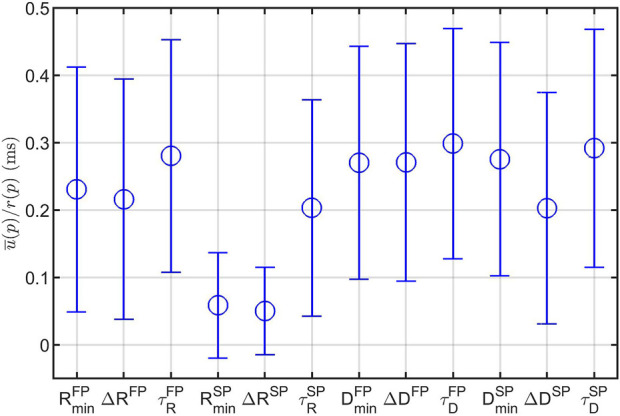
Mean (circles) ± one standard deviation (bars) of the parameter uncertainty estimates *u*(*p*) over all recordings and all patients, normalized with the parameter ranges *r*(*p*). Note that the model parameters $R_{\min}^{SP}$ and Δ*R* ^*SP*^ have a lower uncertainty, indicating a higher impact on the resulting model outcome.

The average *u*(*p*), as explained in Eq. 7, normalized with *r*(*p*), are shown in Figure 6. From this, it is evident that the model parameters relating to the SP are more robustly estimated than their FP counterpart, and that the model parameters relating to the refractory period are more robustly estimated than their conduction delay counterpart. Most noteworthy is the lower uncertainty for $R_{\min}^{SP}$ and Δ*R* ^*SP*^, suggesting a higher impact on the output of the model.

The uncertainty estimates, *u*(*p*), for one patient are shown as red background for each $\hat{\theta }\left(i\right)$ in Figure 5, where again *u*(*p*) for the refractory parameters in the SP is lower. There is also a clear difference in *u*(*p*) between nighttime and daytime, where the uncertainty is much lower at night.

### 3.2 Circadian variation

In Figure 5 we also show an example of the circadian variation (blue lines) for the aforementioned patient, as explained in Eqs. 8, 9, and 10, where a clear distinction between night and day can be seen for most parameters. The average RMSE for the 12 model parameters seen in Figure 5 is 0.22, which can be compared with the average RMSE for all patients and treatment of 0.16 ± 0.03 (mean ± std).

The mean and standard deviation of the circadian variation phase *ϕ* was 1.03 ± 0.74 rad; corresponding to an extreme value at approximately 04:00 am ± 2.8 h.

**FIGURE 7:**
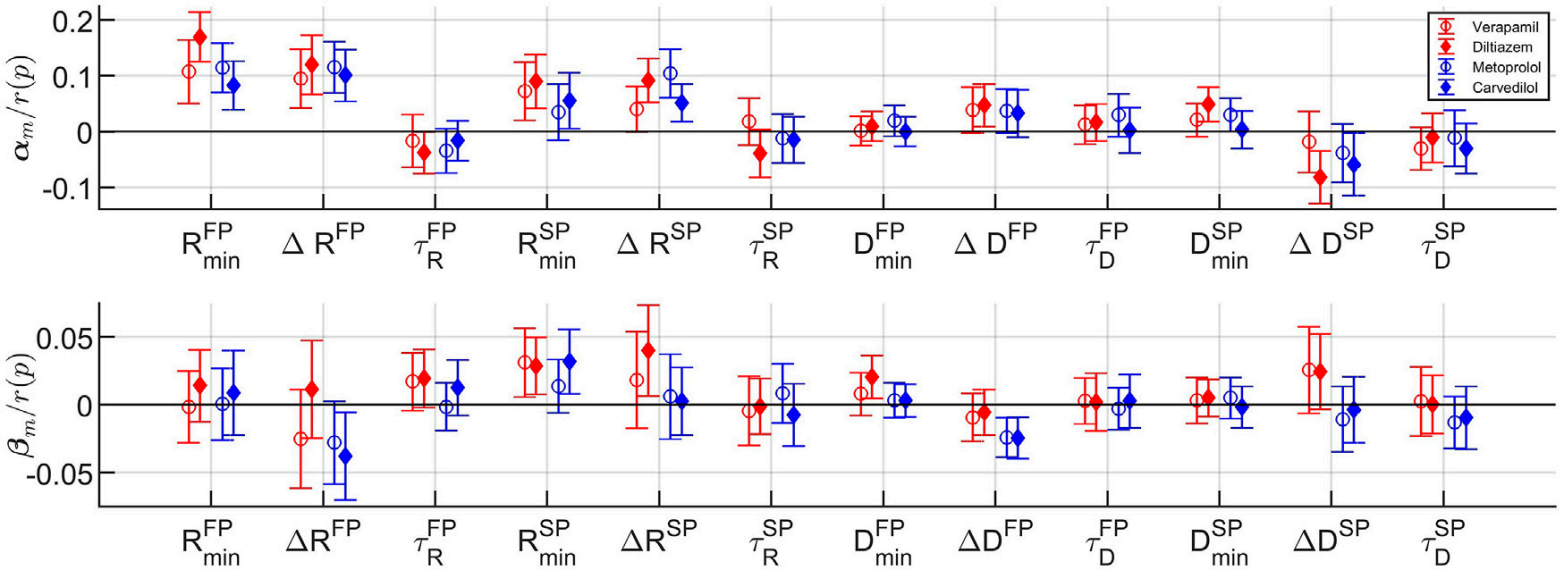
The fixed effects with corresponding 95% confidence intervals for the cosinor mean ***m*** (top) and cosinor amplitude ***a*** (bottom) for each model parameter (cf. Table 1) and drug. Confidence intervals not overlapping zero indicate significant difference from baseline (*p* <0.05).

The fixed-effects ***α***_*m*_ and ***β***_*m*_ and their respective 95% confidence interval, normalized with *r*(*p*), are shown in Figure 7, where the fixed-effects represent the average difference in effect with respect to baseline that the drugs have on the population. It is evident from ***α***_*m*_ in Figure 7 (top panel) that all rate control drugs on average increase the refractory period in both pathways; with a significant increase (*p* < 0.05) in $R_{\min}^{FP}$ and Δ^*FP*^ for all drugs, in $R_{\min}^{SP}$ for all but metoprolol, and in Δ*R* ^*SP*^ for all but verapamil. Moreover, differences between the *β*-blockers and the calcium channel blockers can be observed. Most noticeably for the amplitude (***β***_*m*_) of Δ*D* ^*FP*^ and Δ*D* ^*SP*^, where the two *β*-blockers have a distinctly negative effect in comparison with the two calcium channel blockers.

Detailed results for the estimated fixed and random effects can be found in the Supplementary Material, Section 2.

## 4 Discussion

In this study, we have presented a mathematical framework able to continuously estimate model parameters representing the conduction delay and refractory period of the AV node during 24 h for patients with permanent AF from ECG data. Trends in the estimated model parameters were analyzed using a mixed-effects model to study the circadian variation, where drug-dependent differences could be seen.

The model has previously been shown to be able to represent measured data in the form of histograms and Poincaré plots for 20-min long segments Karlsson et al. (2021). However, continuously estimating model parameters representing the refractory period and conduction delay in the AV node has previously not been possible. A previous study of the RR interval series has indicated that one interval delay in the autocorrelation gives sufficient information to replicate the dynamics of the RR interval series Karlsson et al. (2021). Hence, the Poincaré plot was chosen as a basis for the fitness function in order to take the one interval delay of the RR interval series into account, something that is not possible with an one-dimensional distribution representation such as the histogram. Moreover, since the model describes the impulses from the atria as a stochastic process, it is not possible to have a beat-to-beat level of detail in the fitness function, as evident by the RR interval series in Figure 4.

The choice of segment length N is a trade-off between robustness and time-resolution. The segment length N was set to 1000 RR intervals, corresponding to a time duration of 11 : 53 ± 03 : 28 (mm:ss), to capture changes in RR series characteristics on this time-scale while allowing sufficient estimation accuracy. As a consequence of the choice of N = 1000, the bin size of 50 m was used for the Poincaré plot-based error function. A smaller bin size would allow a more detailed match between model output and data, but would require more RR intervals.

From Figure 4, it is evident that the model and workflow can replicate the histogram and Poincaré plot of obtained RR interval series even for the case with the 95% highest *ϵ*. This was made possible by using the problem-specific GA presented in Section 2.4. Evolutionary algorithms - such as GA - and particle swarm optimization are the most common optimization algorithms used for solving dynamic optimization problems Yazdani et al. (2021); Mavrovouniotis et al. (2017).

One of the main challenges with dynamic optimization problems is the balance between exploration and exploitation, i.e., between searching for different promising regions of the search space, or searching for the optimal solutions within an already promising region. To keep a good level of exploration, the diversity in the population - usually defined as the average Euclidean distance between the individuals in the population - is often monitored. Thus, diversity loss is one of the most critical challenges Yazdani et al. (2021). A great number of methods have been developed to address this diversity loss, often based on randomizing individuals in the population that are too similar to others. For example, crowding - letting new individuals replace the most similar individual in the population Kordestani et al. (2014) - or based on the age of the individuals Das et al. (2013). For GA, it is also possible to combat diversity loss by regulating the mutation rate. However, maintaining a good level of exploration using diversity does not take any information about the data into account. In contrast, changing the mutation rate, the number of immigrants, and the number of generations per segment using Δ*P*(*i*) - as was done in this study - takes information about the data directly into account. Additionally, the number of immigrants in the proposed GA ranges from 10–70%, which limits the initialization’s effect on the overall results. Moreover, the results in Figure 3 indicate that the proposed problem-specific optimization method yields a better fit compared to the standard approach when the characteristics of the data change rapidly. On the other hand, when the characteristics of the data change slowly, the performance is similar even though the proposed algorithm is using fewer generations per segment. The number of RR intervals simulated with the model for each parameter set, *N*_*sim*_, was set to 1500 in the GA based on a trade-off between computational complexity and variation based on the stochastic input sequence to the model. A simulation study relating the variation in *ϵ* and *N*_*sim*_ which was used to guide the decision is shown in the Supplementary Material, Section 1. Moreover, the thresholds for Δ*P* to determine how many generations are to be run per data segment were set so that approximately 55% are run for 1 generation, 30% are run for 2 generations, and the remaining 15% are run for 3.

A variation of Sobol’s method was used to estimate the contribution to output variance for each model parameter, which was related to an increase in error by 10%. This more complex methodology was preferred over a one-at-a-time approach due to the large effect that interaction between model parameters has on the model output. Note that, unlike more traditional uncertainty estimates, this is not directly connected to a probability, since the error function used does not have a proper probabilistic interpretation. Thus, the uncertainty shall only be interpreted as a relative measure between the model parameters, between patients, and between the time of day. For example, it is evident in Figure 5 that the uncertainty for this patient is much lower during nighttime than daytime.

A linear mixed-effect model based on a cosinor analysis was used to derive the circadian variations. This method was used to quantify the circadian variation for the different drugs over the whole population, as well as the individual response to the drugs. The focus of this study is on the population effects of the different drug types in order to understand the drug-dependent differences in the conduction properties, something that needs to be understood before the method could be applicable on an individual level. Even though the focus of this study is on the population level, the individual responses are still of interest, especially for future work. For example, to predict individual responses to different drugs. As shown in Figure 5, the individual match is not optimal, thus a better tool for capturing the circadian variation is believed to be needed before robust analysis on an individual level is feasible. However, the cosinor analysis is an established model for characterizing circadian variation and has previously been used on the RATAF data-set to study heart rate variation Corino et al. (2015a).

From Table 3, in the parameters $R_{\min}^{FP}$ and Δ*R* ^*FP*^, we see a significantly increased refractory period relative to baseline in the FP for all four drugs. In addition, a significant increase in the SP for either $R_{\min}^{SP}$ , or Δ*R* ^*SP*^ could also be seen for all drugs. This increase is also visible in the fixed effect parameters ***α***_*m*_ in Figure 7, top panel. Electrophysiological studies of the two calcium channel blockers verapamil and diltiazem as well as the *β*-blocker metoprolol have shown that the drugs increase the refractoriness in the AV node Leboeuf et al. (1989); Talajic et al. (1992); Rizzon et al. (1978). Moreover, carvedilol has been shown to increase the effective refractory period in the atria during AF Kanoupakis et al. (2004). However, to the best of our knowledge, no studies have been conducted to determine the effect of carvedilol specifically for the refractory period in the AV node. Furthermore, conduction properties in the atria influence the model through the mean arrival rate *λ*, and thus affect the estimated parameters.

In addition, from Figure 7 bottom panel, it is shown that the two *β*-blockers reduce the circadian variation of the conduction delay more than the calcium channel blockers, as evident by Δ*D* ^*FP*^ and Δ*D* ^*SP*^. Stimulation of the *β*_1_-receptors - regulated by the autonomic nervous system - have been shown to increase the conduction velocity in the AV node Gordan et al. (2015). Hence, blocking this receptor using *β*-blocking drugs might decrease the autonomic nervous system effect, and thus reduce the circadian variation, yielding the presented results.

Also seen in Figure 7, the model parameters for the two *β*-blockers often behave similarly. However, the model parameters for the calcium channel blockers verapamil and diltiazem do not always align. In fact, the values for ***α***_*m*_ and ***β***_*m*_ for verapamil are in several cases - most noticeably for $R_{\min}^{FP}$ for ***α***_*m*_ and Δ*R* ^*FP*^, Δ*R* ^*SP*^, and $D_{\min}^{FP}$ for ***β***_*m*_ - similar to those of the two *β*-blockers. Interestingly, it has previously been proposed that the pharmacological effects of verapamil may partly be due to some degree of *β*-blockade Drici et al. (1993).

Moreover, the large confidence intervals in Figure 7, where the majority includes zero, are most likely due to the high inter-patient variability in parameter values. A confidence interval that includes zero would indicate that there is no significant difference from baseline. The high inertia and simplicity of the cosine are other factors in this. For example, some patients have more than one section with parameter values close to those during the night - possibly due to periods of sleep during the day - which a cosine with a period of 24 h could not capture.

### 4.1 Study limitations and future perspectives

The present model of the AV node accounts for dual pathway physiology and rate dependent changes in conduction delay and refractoriness and can simulate retrograde conduction. However, it is not able to simulate some physiological interesting phenomena such as AV node re-entry.

A limited range for the model parameters was used to make the optimization more efficient. The choice of the boundaries was guided by electrophysiological measurements from previous clinical studies while keeping a conservative range to not exclude realistic values. The maximal refractory period for the model - given as the sum of *R*_min_ and Δ*R* - lies in the range [150, 1350] ms and was set to include the effective refractory period of the AV node, which has been reported as 361 ± 57 and 283 ± 48 m for the FP and SP, respectively Natale et al. (1994). Furthermore, the conduction delay of the whole model is given by the sum of *D*_min_ and Δ*D* multiplied by the number of nodes, which lies in the range [0, 1050] ms. Thus, it includes all realistic PR intervals, which rarely exceed 200 m Schumacher et al. (2017). Even though the boundaries were conservatively chosen, we cannot exclude the possibility that a different choice would have affected the resulting parameter values. Moreover, since the parameters might be hard to interpret, combining the model parameters associated with the same conduction property, i.e., the two refractory periods and the two conduction delays, to create more interpretable representations, is interesting.

As mentioned before, high inertia and simplicity of the cosine are limiting factors for the assessment of circadian variation. However, the cosinor analysis is an established model for characterizing circadian variation and is thus important for clear and interpretable results. Using the estimated uncertainty to weight the estimated parameters is one possible approach to make the cosine fit the estimates better. Other methods to capture the differences in the AV node parameters over time, such as time-frequency analysis of the estimated parameter trends, should also be investigated.

It should be noted that the estimated model parameters are not clinically validated for assessment of AV node refractoriness and conduction delay. Hence, the clinical significance of the results should be interpreted with caution. However, the overall findings for the different drugs on the whole population are, as discussed above, in accordance with electrophysiological studies. Another limitation is the sample size of 60 patients in combination with the high inter-patient variability in parameter values, as seen in the large standard deviation in Table 3. This makes the population estimates more uncertain, partly causing the large confidence intervals seen in Figure 7.

A natural continuation of this work is to analyze the estimated model parameters during baseline, possible in combinations with other factors such as age or gender, to predict the mean heart rate under the influence of the different drugs. This in turn could be used to assist in personalized treatment selection during AF.

## 5 Conclusion

We have presented a framework - including a mathematical model and a genetic algorithm - which for the first time enables characterization of the refractory period and the conduction delay of the AV node during 24 h for patients with permanent AF, solely based on non-invasive data.

With ECG from AF patients during baseline and under the influence of different rate control drugs, a mixed-model framework was used on the estimated model parameters to compare the impact on circadian variation between drugs. From this, differences in conduction delay could be identified between *β*-blockers and calcium channel blockers, which was previously unknown.

## Data Availability

The data analyzed in this study is subject to the following licenses/restrictions: The estimated model parameters **θ_(i)_** and associated uncertainty estimate u(p) supporting the conclusions for this article will be available from MK upon request. The ECG data are owned by Vestre Viken Hospital Trust, and requests for access can be made to SU. The code for the model together with an user example can be found at https://github.com/FraunhoferChalmersCentre/AV-node-model.
