# Supplementary material for: ECG based assessment of circadian variation in AV-nodal conduction during AF—Influence of rate control drugs
Source: Front Physiol. 2022 Oct 4;13:976526. doi: 10.3389/fphys.2022.976526 (PMC9577140; doi:10.3389/fphys.2022.976526)
Supplement: Supplementary file 1 [file DataSheet1.PDF]

Supplementary Material

This supplementary material is divided into two sections. Section 1 covers details about the genetic algorithm (GA) used to estimate $\hat{\boldsymbol{\theta}}(i)$, and section 2 includes detailed results for the linear mixed-effect model.

1 GENETIC ALGORITHM

To generate the next generation from the current one, we utilize tournament selection to select parents, two-point crossover for generating offsprings, and creep mutation for adding genetic variability to the new generation. The algorithm is dependent on five hyper-parameters, which are functions of the Poincaré difference ΔP, described in Equation 5 of the main article. Below we give a detailed description of the procedure and the computation of the hyper-parameters.

The GA starts by initializing the 400 individuals ($\boldsymbol{\theta}$) in the population randomly using a latin hypercube sampling in the ranges: $\{R_{min}^{SP}, R_{min}^{FP}\} \in [150, 650]\ ms$; $\{\Delta R^{SP}, \Delta R^{FP}\} \in [0, 700]\ ms$, $\{\tau_R^{SP}, \tau_R^{FP}\} \in [40, 300]\ ms$; $\{D_{min}^{SP}, D_{min}^{FP}\} \in [0, 30]\ ms$; $\{\Delta D^{SP}, \Delta D^{FP}\} \in [0, 75]\ ms$; $\{\tau_D^{SP}, \tau_D^{FP}\} \in [40, 300]\ ms$. All 400 individuals are then evaluated by running the model and comparing the model output to the current data, as explained by Equation 4 in the main article.

Two individuals are selected with uniform probability for all individuals, and the fittest of these two are picked with a probability of 0.7. This procedure is done again, resulting in a selection of two different individuals. This is commonly called tournament selection. The two selected individuals are then, with a probability of $P_C(\Delta P)$, combined using a two-point crossover, meaning that two crossover points are picked randomly on the parameter vector and the sections in between the two points are swapped between the selected individuals to create two new individuals Wahde (2008). Otherwise, the two selected parameters skip the crossover phase. The two new individuals, either newly created by crossover or the originally selected individuals, are then with a probability of $P_M^{Ind}(\Delta P)$ selected for mutation. Each parameter for an individual that is selected for mutation will with a probability of $P_M^P(\Delta P)$ be mutated. The mutation is created by creep mutation, i.e., by adding a number drawn from a zero-mean normal distribution with standard deviation $r(p)\sigma_M(\Delta P)$ to the individual's parameter, where $r(p)$ is the range of the selected parameter p, as defined in the main article. This procedure from selection of parents to the creation of two new individuals is performed until there are 390 new individuals, before the ten most fit individuals from the previous generation are added. This new population of 400 does then replace the previous one.

The fitness value of the newly created 400 individuals is then evaluated, before randomly selecting a number of individuals, $N_I(\Delta P)$, from the 300 least fit individuals to be replaced by immigrants. One third of the immigrants are created by latin hypercube sampling, in the same ranges as the initialization. One third is selected by simultaneously running eight computationally faster GA, with the same structure and hyper-parameters as the main GA but using only 16 individuals, where the fittest ones are selected for immigration. The last third of the immigrants are created from a saved-up memory of previously individuals, where the ten fittest individuals from each generation as well as their model output, λ, and RR_{min} are saved. From this memory, the ten previously generations with the most similar λ and RR_{min} are first selected, evaluated by the combined difference $\Delta C(\Delta\lambda, \Delta RR_{min})$ seen in Equation S1,

$$\Delta C(\Delta\lambda, \Delta RR_{min}) = \frac{|\Delta\lambda|}{6} + \frac{|\Delta RR_{min}|}{300}, \tag{S1}$$

where $\Delta\lambda$ and ΔRR_{min} are the difference between the current λ and RR_{min}, respectively, and the ones saved in the memory. From these ten selected generations, the individuals with the highest fitness computed using the current RR interval series are used for immigration.

After immigration, which only occurs at the first generation the GA runs for a given data segment, the process of selection, crossover, and mutation starts over again. The number of generations the GA runs before switching to the next data segment varies depending on ΔP, from 1 when $\Delta P < 800$; to 2 when $800 \leq \Delta P < 2000$; to 3 when $\Delta P \geq 2000$. Furthermore, the five hyper-parameters $\boldsymbol{H} = \{P_C, P_M^{Ind}, P_M^P, \sigma_M, N_I\}$ are also tuned using ΔP, according to Equation S2,

$$\begin{aligned}
&\boldsymbol{H}_{Min}\; if\; \Delta P < 400, \\
&\boldsymbol{H}_{Min} + \frac{\boldsymbol{H}_{Mean} - \boldsymbol{H}_{Min}}{1000 - 400}(\Delta P - 400)\; if\; 400 < \Delta P < 1000, \\
&\boldsymbol{H}_{Mean} + \frac{\boldsymbol{H}_{Max} - \boldsymbol{H}_{Mean}}{5000 - 1000}(\Delta P - 1000)\; if\; 1000 < \Delta P < 5000, \\
&\boldsymbol{H}_{Max}\; if\; 5000 < \Delta P.
\end{aligned} \tag{S2}$$

Thus, the hyper-parameters dependence on ΔP are non-linear. The values for $\boldsymbol{H}_{Min}$, $\boldsymbol{H}_{Mean}$ and $\boldsymbol{H}_{Max}$ for the different hyper-parameters are given in Table S1.

Table S1. Ranges for the hyper-parameters that change depending on ΔP. All ranges are empirically determined.

Parameter	Minimum, $\boldsymbol{H}_{Min}$	Mean, $\boldsymbol{H}_{Mean}$	Maximum, $\boldsymbol{H}_{Max}$
P_C	0.4	0.55	0.7
P_M^{Ind}	0.6	0.75	0.9
P_M^P	$\frac{1}{12}$	$\frac{2}{12}$	$\frac{5}{12}$
σ_M	0.025	0.06	0.12
N_I	0.10	0.25	0.7

1.1 Simulation study for determining N_{sim}

To determine the value for the number of simulated RR intervals, N_{sim}, a simulation study was completed. A low N_{sim} would make the simulated RR interval series vary more between realizations due to the stochastic input sequence from the Poisson process, whereas a high N_{sim} would increase the computational complexity. Thus, the simulation study analyses the relation between N_{sim} and the variation between realizations by simulating a RR interval series with a fixed $\boldsymbol{\theta}$, λ, and RR_{min}. By running the model 10000 times using the fixed values and calculating the error ϵ using Equation 4 in the main article, an estimate of how much the variation between realizations changes depending on N_{sim} was created. This is visualized in Figure S1.

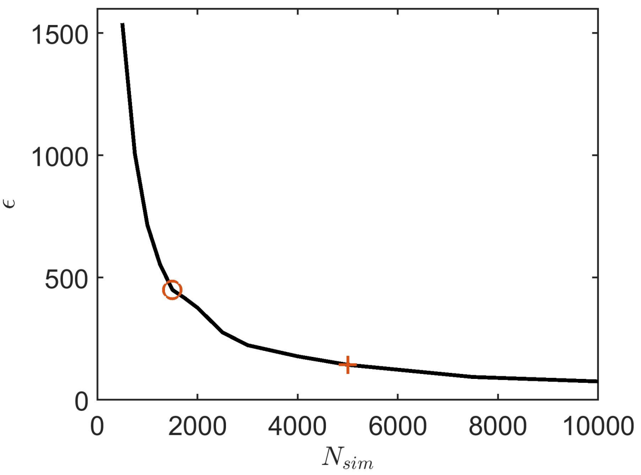

Figure S1. A simulation study of the relation between variation between realizations and N_{sim}. The red circle marks 1500 which was used for the bulk of the GA, and the red cross marks 5000 which was used for the ten best individuals in each generation. The model parameter values used were; $R^{FP}_{min} = 275$, $\Delta R^{FP} = 175$, $\tau^{FP}_{R} = 105$, $R^{SP}_{min} = 275$, $\Delta R^{SP} = 175$, $\tau^{SP}_{R} = 105$, $D^{FP}_{min} = 7.5$, $\Delta D^{FP} = 18.75$, $\tau^{FP}_{D} = 100$, $D^{SP}_{min} = 7.5$, $\Delta D^{SP} = 18.75$, and $\tau^{SP}_{D} = 100$.

2 DETAILED RESULTS FOR THE LINEAR MIXED-EFFECT MODEL

The fixed-effect for the linear mixed-effect model described in Equations 8, 9, and 10 in the main article are shown in Table S2, where subscript V indicates verapamil, D indicates diltiazem, M indicates metoprolol, and C indicates carvedilol. Moreover, boxplots of the random-effects are shown in Figure S2.

Table S2. The estimated fixed effect for the linear mixed-effect model.

Fixed effect	R^{FP}_{min}	ΔR^{FP}	τ^{FP}_{R}	R^{SP}_{min}	ΔR^{SP}	τ^{SP}_{R}	D^{FP}_{min}	ΔD^{FP}	τ^{FP}_{D}	D^{SP}_{min}	ΔD^{SP}	τ^{SP}_{D}
$\boldsymbol{\alpha}(ms)$	434	408	172	241	237	180	5.3	19	141	21	26	187
$\boldsymbol{\beta}(ms)$	41	88	16	22	69	22	1.1	5.7	15	1.5	6.4	20
$\boldsymbol{\alpha}_V(ms)$	54	67	-4.3	36	28	4.7	0.03	2.9	3.0	0.63	-1.4	-7.3
$\boldsymbol{\alpha}_D(ms)$	85	84	-9.8	45	64	-10	0.29	3.5	4.0	1.5	-6.1	-2.6
$\boldsymbol{\alpha}_M(ms)$	58	81	-8.9	17	73	-3.1	0.59	2.8	7.1	0.90	-2.9	-2.8
$\boldsymbol{\alpha}_C(ms)$	41	71	-4.2	27.5	35.9	-3.8	0.00	2.5	0.6	0.10	-4.4	-7.3
$\boldsymbol{\beta}_V(ms)$	-0.8	-17	4.5	15.6	12.8	-1.2	0.24	-0.70	0.68	0.10	1.9	0.59
$\boldsymbol{\beta}_D(ms)$	7.1	8.0	5.1	14	28	-0.31	0.62	-0.42	0.46	0.16	1.8	0.06
$\boldsymbol{\beta}_M(ms)$	0.26	-19	-0.39	6.9	4.2	2.2	0.10	-1.8	-0.69	0.15	-0.80	-3.1
$\boldsymbol{\beta}_C(ms)$	4.4	-26	3.3	15	1.8	-1.9	0.09	-1.8	0.67	-0.05	-0.27	-2.3

REFERENCES

Wahde, M. (2008). *Biologically inspired optimization methods: an introduction* (WIT press)

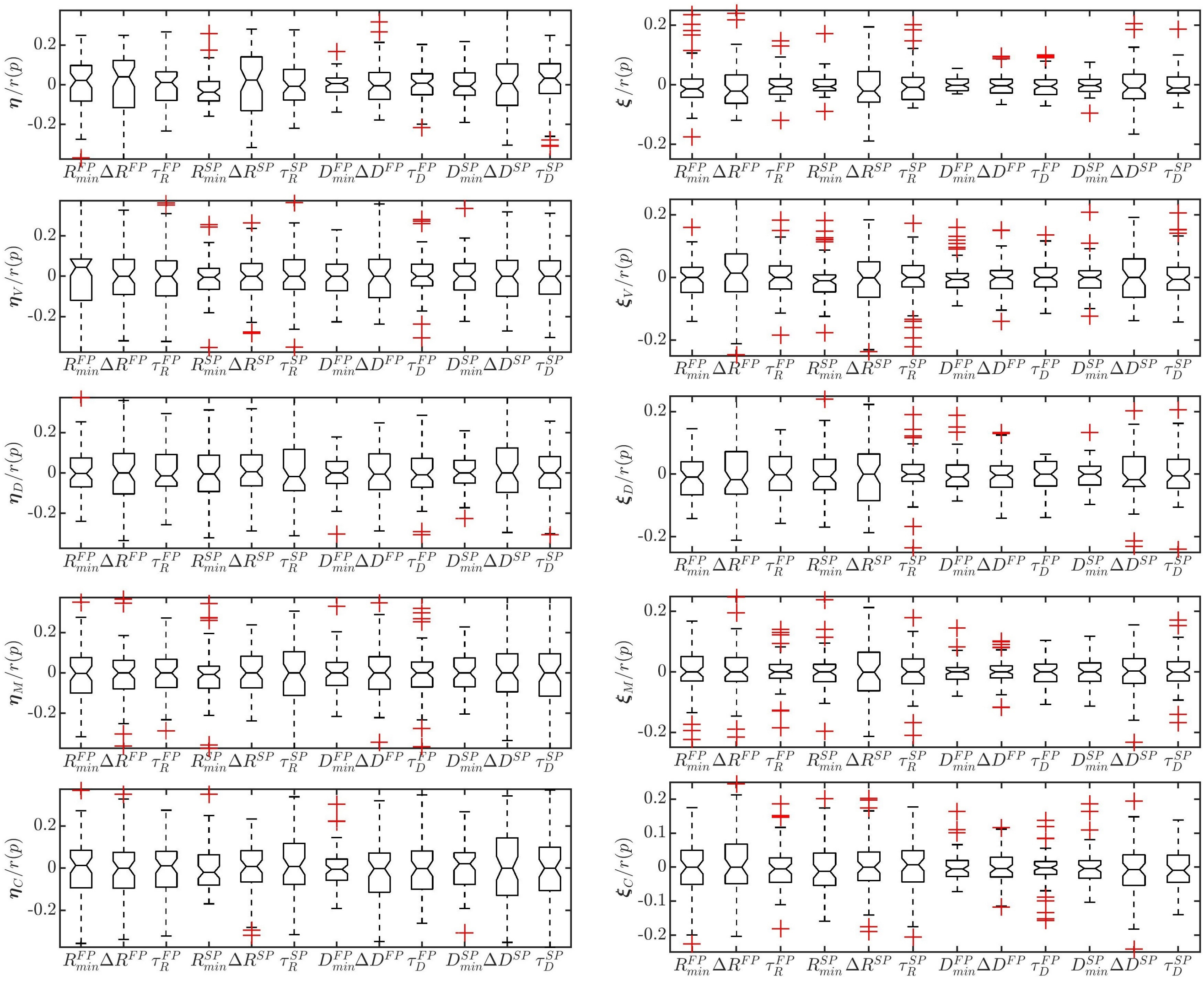

Figure S2. Boxplots of the random-effect normalized with the range for each parameter $r(p)$, for the the cosine mean $\boldsymbol{m}$ (left) and the amplitude $\boldsymbol{a}$ (right) in the linear mixed-effect model. On each box, the central mark indicates the median, and the bottom and top edges of the box indicate the 25th and 75th percentiles, respectively. The whiskers mark the most extreme data points not considered outliers, and the outliers are plotted with a red '+'.
